# Risk factors for erlotinib-induced hepatotoxicity: a retrospective follow-up study

**DOI:** 10.1186/s12885-018-4891-7

**Published:** 2018-10-16

**Authors:** Min Kyoung Kim, Jeong Yee, Yoon Sook Cho, Hong Won Jang, Ji Min Han, Hye Sun Gwak

**Affiliations:** 1 Graduate School of Converging Clinical & Public Health, Ewha Womans University, Seoul, 03760 Korea; 2 Department of Pharmacy, Seoul National University Hospital, Seoul, 03080 Korea; 3 College of Pharmacy & Division of Life and Pharmaceutical Sciences, Ewha Womans University, 52 Ewhayeodae-gil Seodaemun-gu, Seoul, 03760 Republic of Korea

**Keywords:** Erlotinib, Hepatotoxicity, CYP3A4 inducers, H2-antagonist, Proton pump inhibitor

## Abstract

**Background:**

Erlotinib is a drug used for the treatment of non-small cell lung cancer (NSCLC) and pancreatic cancer. Severe hepatotoxicity was observed in 4% to 31% of patients receiving erlotinib treatment prompting delay or termination of treatment. Only a few factors related to hepatotoxicity of erlotinib have been reported. No study has investigated the role of concomitant medications and erlotinib-induced hepatotoxicity. The aim of this study was to investigate the association between erlotinib-induced hepatotoxicity and various factors including concomitant medications in patients with NSCLC and pancreatic cancer.

**Methods:**

From January 2014 to June 2017, a retrospective study was conducted in patients with NSCLC and pancreatic cancer, who were treated with erlotinib. Various data were reviewed, including sex, age, body weight, height, body surface area (BSA), underlying disease, Eastern Cooperative Oncology Group (ECOG) Performance Status (PS), smoking history, erlotinib dose, EGFR mutation, and concomitant drugs.

**Results:**

The incidence of grade 2 or higher hepatotoxicity in the study group of patients was 17.2%. Multivariate analysis showed a 2.7-fold increase in hepatotoxicity with the concomitant use of CYP3A4 inducers. In NSCLC patients, co-administration of H2-antagonist/PPI increased hepatotoxicity 3.5-fold. Among the demographic factors, liver metastasis and age ≥ 65 years were significant risk factors in all study patients and NSCLC patients, respectively; the attributable risks for liver metastasis and age were 46.3% and 71.8%, respectively. Subgroup analysis using pancreatic cancer patients yielded marginally significant results with CYP3A4 inducers and erlotinib-induced hepatotoxicity. Liver metastasis and CYP3A4 inducers also shortened time to hepatotoxicity 2.1 and 2.3-fold, respectively.

**Conclusions:**

Our study showed that concomitant use of CYP3A4 inducers and H2-antagonist/PPI, liver metastasis, and age ≥ 65 were associated with erlotinib-induced hepatotoxicity. Thus, close monitoring of liver function is recommended, especially in patients using CYP3A4 inducers and anti-acid secreting agents.

## Background

The reported rates of incidence of lung cancer and pancreatic cancer in Korea are 11.1 and 2.7%, respectively. These cancers are fatal with 5-year survival rates of 25.1% and 10.1%, respectively [1]. Lung cancer and pancreatic cancer often overexpress epidermal growth factor receptor (EGFR), which is associated with a worse prognosis [2, 3].

Erlotinib, an EGFR tyrosine kinase inhibitor (TKI), is used for the treatment of non-small cell lung cancer (NSCLC) and pancreatic cancer. Erlotinib has been shown to prolong survival and decrease symptoms compared with placebo in previously treated patients with NSCLC [4]. Studies also showed that erlotinib improved progression-free survival compared with chemotherapy as a first-line treatment in Asian patients with NSCLC carrying activating EGFR mutations [5]. Patients with advanced pancreatic cancer show poor diagnosis, and gemcitabine monotherapy fails to improve survival rate. The addition of EGFR TKIs such as erlotinib to gemcitabine demonstrated a significantly higher survival rate in patients diagnosed with pancreatic cancer [3].

Common toxicities associated with erlotinib are mostly mild in severity and manageable, and include skin rash, diarrhea, and nausea [6]. Hepatotoxicity involving elevated liver transaminase level grade 2 or higher according to the Common Terminology Criteria for Advanced Events (CTCAE), Version 4.0 has been observed in 31% of pancreatic cancer patients and 4% NSCLC patients receiving erlotinib treatment [3, 6]. Severe hepatotoxicity is not frequent; however, patients who experience hepatotoxicity while receiving erlotinib often need to postpone or terminate treatment.

The cause of erlotinib-induced hepatotoxicity is unknown. Erlotinib is metabolized in the liver mainly by cytochrome P450 (CYP)3A4 and minimally by CYP1A1 and CYP1A2. *O*-desmethylated erlotinib, the major metabolite, is found in human plasma [7]. Cytolytic hepatitis is caused by several factors including toxic metabolic intermediates, autoimmune injury, and direct EGFR TKI inhibition [8]. However, studies investigating the toxicity mechanisms mainly involved gefitinib-induced hepatotoxicity. Only a few cases of erlotinib-induced hepatotoxicity have been reported. The role of concomitant medications and erlotinib-induced hepatotoxicity has not been studied. Therefore, the aim of this current study was to investigate the association between erlotinib-induced hepatotoxicity and various factors including concomitant medications in pancreatic cancer and NSCLC patients.

## Methods

### Patients

From January 2014 to June 2017, a retrospective study was performed with patients who were older than 18 years and treated with erlotinib at Seoul National University Hospital, Korea. The exclusion criteria were: patients who were not diagnosed with NSCLC or pancreatic cancer, had underlying liver diseases (fatty liver, alcoholic liver cirrhosis, and hepatitis), an elevated aspartate aminotransferase (AST) or alanine aminotransferase (ALT) level on day one of erlotinib administration, and lack of liver function test results. This study was approved by the Seoul National University Hospital Institutional Review Board (IRB # H-1710-087-894).

The following data were collected: sex, age, body weight, height, body surface area (BSA), underlying disease, Eastern Cooperative Oncology Group (ECOG) Performance Status (PS), smoking history, erlotinib dosage, EGFR mutations, and concomitant drug usage. Concomitant drugs included CYP3A4 inhibitors, CYP3A4 inducers, CYP2D6 inhibitors, H2-antagonists, and proton pump inhibitors (PPI). The CYP3A4 inhibitors included amiodarone, aprepitant, atazanavir, cimetidine, ciprofloxacin, clarithromycin, danazol, diltiazem, fluconazole, fluoxetine, fluvoxamine, imatinib, itraconazole, lapatinib, nicardipine, nifedipine, ritonavir, verapamil, and voriconazole. The CYP3A4 inducers included carbamazepine, dexamethasone, efavirenz, ethosuximide, etravirine, naficillin, oxcarbazepine, phenobarbital, phenytoin, primidone, rifabutin, and rifampicin (rifampin). The H2-antagonists included cimetidine, famotidine, nizatidine, and ranitidine. The PPIs included (es)omeprazole, (dex)lansoprazole, pantoprazole, and rabeprazole.

### Erlotinib administration and laboratory assessment

Patients with NSCLC were treated with an erlotinib dose of 150 mg and those diagnosed with pancreatic cancer were adminstered 100 mg of the same drug. Gemcitabine was prescribed for patients with pancreatic cancer at a dose of 1000 mg/m^2^. Liver function was tested at 2–3 weeks after erlotinib therapy initially, and every 2 to 3 months thereafter. Serum ALT and AST levels were evaluated. The hepatotoxicity grade was determined using the Common Terminology Criteria for Adverse Events (CTCAE), Version 4.0. The CTCAE defines grades I, II, III, and IV toxicity levels of AST and ALT as 1.0–3.0 times, 3.0–5.0 times, 5.0–20.0 times, and more than 20 times the upper limit of normal, respectively. In this study, hepatotoxicity was defined as grade II or higher.

### Statistical analysis

The chi-squared test or Fisher’s exact test were used to compare the categorical variables between patients with and without hepatotoxicity. Multivariable logistic regression analysis was used to identify independent risk factors for hepatotoxicity. Factors with *p*-values < 0.1 from univariate analysis along with strong confounders such as sex were included in multivariate analysis. Odds ratio (OR) and adjusted odds ratio (AOR) were calculated from univariate and multivariate analyses, respectively. Attributable risk was calculated by 1–1/OR. The time to hepatotoxicity and recovery was analyzed using the Kaplan-Meier survival curves and the log-rank test. Cox’s proportional-hazards model was used for multivariate analysis. Hazard ratio (HR) and adjusted hazard ratio (AHR) were calculated from univariate and multivariate analyses, respectively. *P*-values less than 0.05 were considered statistically significant. All statistical analyses were carried out using the Statistical Package for Social Sciences (SPSS) version 17.0 for Windows (Inc., Chicago, IL, USA).

## Results

A total of 448 patients were eligible for participation in the study from January 2014 to June 2017. The following patients were excluded: those who were not diagnosed with NSCLC or pancreatic cancer (*n* = 8), had underlying liver diseases (*n* = 14), had an elevated AST or ALT value on day one of erlotinib administration (*n* = 30), and those without liver function test results (*n* = 36). Accordingly, data from 360 patients were used for the analysis including 155 patients with lung cancer and 205 with pancreatic cancer.

As shown in Table 1, patients’ mean age was 64 years (range 28–86); 177 patients (49.2%) were ≥ 65 years of age. About 40% of the study patients were women. Drugs concurrently administered with erlotinib included CYP3A4 inhibitors (*n* = 18), CYP3A4 inducers (*n* = 22), H2-antagonists (*n* = 50), PPI (*n* = 30), and any of the two anti-acid secreting agents H2-antagonist or PPI (H2-antagonist/PPI, *n* = 72). The incidence of hepatotoxicity was 17.2%; the frequency of hepatotoxicity in patients with lung and pancreatic cancer was 10.3% and 22.4%, respectively.

**Table 1:** Hepatotoxicity related to erlotinib administration

Characteristics	All patients		Lung cancer		Pancreatic cancer	
	Hepatotoxicity No (%)		Hepatotoxicity No (%)		Hepatotoxicity No (%)	
	Presence (*n* = 62)	Absence (*n* = 298)	Presence (*n* = 16)	Absence (*n* = 139)	Presence (*n* = 46)	Absence (*n* = 159)
Age (years)						
< 65	28 (45.2)	155 (52.0)	6 (37.5)	85 (61.2)	22 (47.8)	70 (44.0)
≥ 65	34 (54.8)	143 (48.0)	10 (62.5)	54 (38.8)	24 (52.2)	89 (56.0)
Sex						
Male	35 (56.5)	182 (61.1)	8 (50.0)	87 (62.6)	27 (58.7)	95 (59.7)
Female	27 (43.5)	116 (38.9)	8 (50.0)	52 (37.4)	19 (41.3)	64 (40.3)
BW (kg)						
< 60	38 (61.3)	162 (54.4)	6 (37.5)	70 (50.4)	32 (69.6)	92 (57.9)
≥ 60	24 (38.7)	136 (45.6)	10 (62.5)	69 (49.6)	14 (30.4)	67 (42.1)
Height (cm)^a^						
< 160	27 (43.5)	115 (38.7)	8 (50.0)	45 (32.4)	19 (41.3)	70 (44.3)
≥ 160	35 (56.5)	182 (61.3)	8 (50.0)	94 (67.6)	27 (58.7)	88 (55.7)
BSA^b^						
< 1.6	32 (51.6)	130 (43.8)	5 (31.3)	54 (38.8)	27 (58.7)	76 (48.1)
≥ 1.6	30 (48.4)	167 (56.2)	11 (68.6)	85 (61.2)	19 (41.3)	82 (51.9)
Stage^c^						
1–3	6 (14.3)	27 (14.4)	0 (0.0)	5 (5.0)	6 (20.7)	22 (25.0)
4	36 (85.7)	161 (85.6)	13 (100.0)	95 (95.0)	23 (79.3)	66 (75.0)
CV						
Yes	15 (24.2)	83 (27.9)	1 (6.3)	25 (18.0)	14 (30.4)	58 (36.5)
No	47 (75.8)	215 (72.1)	15 (93.8)	114 (82.0)	32 (69.6)	101 (63.5)
DM						
Yes	18 (29.0)	66 (22.1)	1 (6.3)	17 (12.2)	17 (37.0)	49 (30.8)
No	44 (71.0)	232 (77.9)	15 (93.8)	122 (87.8)	29 (63.0)	110 (69.2)
EGFR mutations^d^						
Yes	9 (81.8)	76 (69.1)	9 (81.8)	76 (69.1)	NA	NA
No	2 (18.2)	34 (30.9)	2 (18.2)	34 (30.9)	NA	NA
Liver metastasis						
Yes	19 (30.6)	61 (20.5)	2 (12.5)	11 (7.9)	17 (37.0)	50 (31.4)
No	43 (69.4)	237 (79.5)	14 (87.5)	128 (92.1)	29 (63.0)	109 (68.6)
CYP3A4 Inhibitor						
Yes	3 (4.8)	15 (5.0)	2 (12.5)	10 (7.2)	1 (2.2)	5 (3.1)
No	59 (95.2)	283 (95.0)	14 (87.5)	129 (92.8)	45 (97.8)	154 (96.9)
CYP3A4 Inducer						
Yes	7 (11.3)	15 (5.0)	3 (18.8)	11 (7.9)	4 (8.7)	4 (2.5)
No	55 (88.7)	283 (95.0)	13 (81.3)	128 (92.1)	42 (91.3)	155 (97.5)
H2 blocker or PPI						
Yes	16 (25.8)	56 (18.8)	7 (43.8)	28 (20.1)	9 (19.6)	28 (17.6)
No	46 (74.2)	242 (81.2)	9 (56.3)	111 (79.9)	37 (80.4)	131 (82.4)
PPI						
Yes	7 (11.3)	23 (7.7)	4 (25.0)	12 (8.6)	3 (6.5)	11 (6.9)
No	55 (88.7)	275 (92.3)	12 (75.0)	127 (91.4)	43 (93.5)	148 (93.1)
H2 blocker						
Yes	9 (14.5)	41 (13.8)	3 (18.8)	22 (15.8)	6 (13.0)	19 (11.9)
No	53 (85.5)	257 (86.2)	13 (81.3)	117 (84.2)	40 (87.0)	140 (88.1)

*BW* body weight, *BSA* body surface area, *ECOG PS* Eastern Cooperative Oncology Group performance scale, *CV* Cardiovascular diseases, *DM* Diabetes Mellitus, *EGFR* epidermal growth factor receptor, *PPI* proton pump inhibitor, *NA* not available

^a^There was 1 missing data for height

^b^There was 1 missing data for BSA

^c^There were 130 missing data for stage

^d^There were 34 missing data for EGFR mutation in lung cancer patients

Multivariate analysis showed that liver metastasis and CYP3A4 inducers increased hepatotoxicity after controlling for variables with *P* value less than 0.1 from univariate analysis; the attributable risk of liver metastasis and CYP3A4 inducers was 46.3% and 62.4%, respectively (Table 2).

**Table 2:** Univariate and multivariate regression analysis to identify predictors for hepatotoxicity related to erlotinib administration

Characteristics	Unadjusted OR (95% CI)	Adjusted OR (95% CI)	Attributable risk
Male	0.826 (0.475–1.437)	0.987 (0.516–1.886)	
Age ≥ 65	1.316 (0.760–2.280)	1.257 (0.718–2.203)	
BSA ≥ 1.6^a^	0.730 (0.422–1.263)	0.728 (0.383–1.385)	
Liver metastasis	1.717 (0.934–3.156)	1.862 (1.001–3.465)^*^	46.3
CYP3A4 Inducer	2.401 (0.936–6.162)	2.660 (1.013–6.982)^*^	62.4

For multivariate analysis, factors with *p* < 0.1 in the univariate analysis were included in addition to sex, age and BSA

*BSA* body surface area

^a^There was 1 missing data for BSA

*P < 0.05

**Fig. 1:**
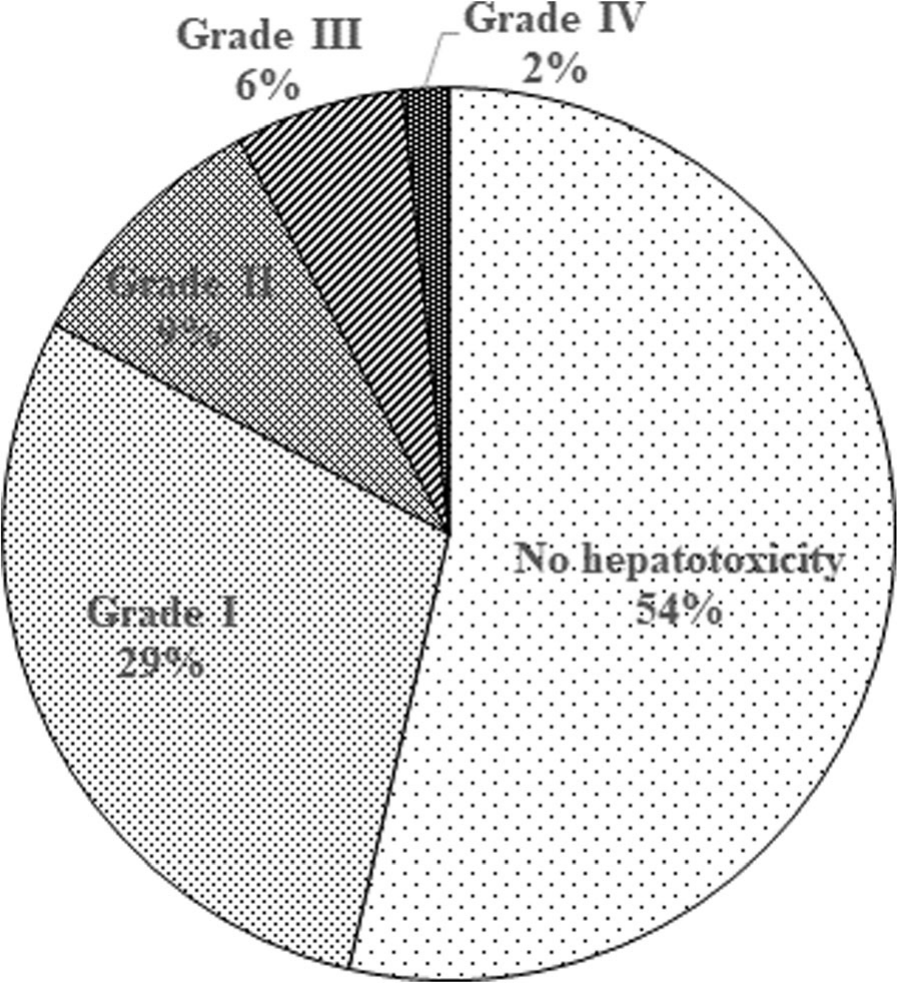
Proportion of patients with maximum CTCAE hepatotoxicity grade (%)

The proportion of patients in each CTCAE hepatotoxicity grade was shown in Fig. 1. Twenty-two patients (6%) and 6 patients (2%) experienced grade III and grade IV hepatotoxicity, respectively. Diabetes mellitus (DM), pancreatic cancer, and liver metastasis were most frequently observed in patients with grade III and IV hepatotoxicity; while DM was a significant factor (AOR 2.3, 95% CI 1.0–5.1), the latter two were of marginal significance. Among 6 patients with grade IV hepatotoxicity, two-third of patients had pancreatic cancer, and the remaining 2 lung cancer patients had EGFR mutation, although there was no statistically significant factor.

For the analysis of lung cancer subgroup (Table 3), two models were constructed since PPIs were included in H2-antagonist/PPI. Model I included PPI, in addition to age, sex, and BSA and Model II included H2-antagonist/PPI instead of PPI in Model I. Age ≥ 65 (AOR 3.2~ 3.5) and H2-antagonist/PPI (AOR 3.5) were significant risk factors for hepatotoxicity after adjusting for confounders. In the subgroup analysis using pancreatic cancer patients, only CYP3A4 inducers showed marginal significance in the multivariate analysis (*p* = 0.055) (Table 4).

**Table 3:** Univariate and multivariate regression analysis to identify predictors for hepatotoxicity related to erlotinib administration in lung cancer patients

Characteristics	Unadjusted OR (95% CI)	Model I		Model II	
		Adjusted OR (95% CI)	Attributable risk	Adjusted OR (95% CI)	Attributable risk
Male	0.598 (0.212–1.688)	0.251 (0.060–1.048)		0.266 (0.065–1.084)	
Age ≥ 65	2.623 (0.902–7.633)	3.198 (1.023–9.997)^*^	68.7	3.540 (1.123–11.164)^*^	71.8
BSA ≥ 1.6	1.398 (0.460–4.244)	3.545 (0.787–15.972)		3.188 (0.723–14.053)	
PPI	3.528 (0.984–12.651)	3.529 (0.916–13.595)			
H2 blocker or PPI	3.083 (1.056–9.000)^*^			3.454 (1.114–10.713)^*^	71.0

For model I construction, sex, age, BSA and PPI were included for analysis. For model II construction, sex, age, BSA and H2 blocker or PPI were included for analysis

*BSA* body surface area, *PPI* proton pump inhibitor

*P < 0.05

**Table 4:** Univariate and multivariate regression analysis to identify predictors for hepatotoxicity related to erlotinib administration in pancreatic cancer patients

Characteristics	Unadjusted OR (95% CI)	Adjusted OR (95% CI)
Male	0.957 (0.491–1.865)	1.279 (0.598–2.737)
Age ≥ 65	0.858 (0.444–1.656)	0.731 (0.369–1.448)
BSA ≥ 1.6^a^	0.652 (0.336–1.268)	0.549 (0.255–1.180)
CYP3A4 Inducer	3.690 (0.886–15.679)	4.114 (0.969–17.465)

For multivariate analysis, factors with *p* < 0.1 in the univariate analysis were included in addition to sex, age and BSA

*BSA* body surface area

^a^There was 1 missing data for BSA

**Fig. 2:**
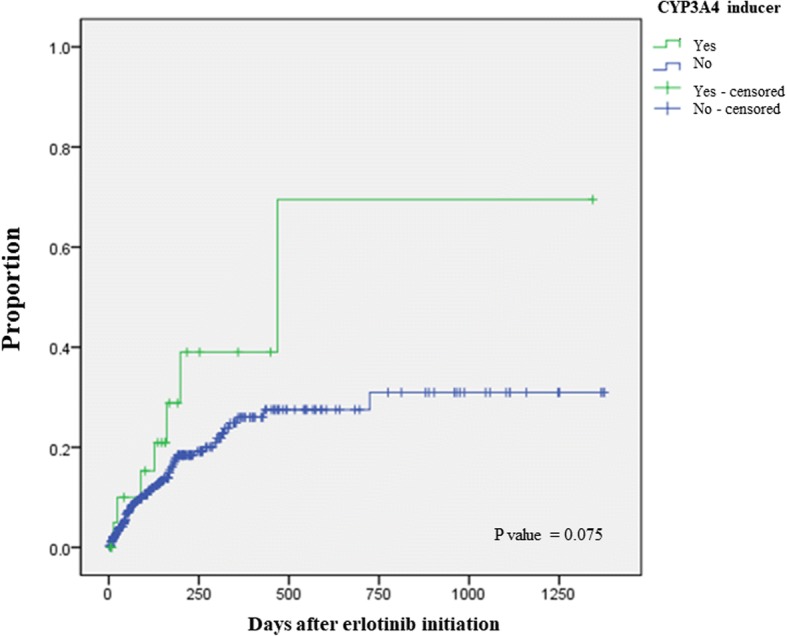
Kaplan-Meier curve of time to erlotinib-induced hepatotoxicity comparing patients with and without concomitant use of CYP3A4 inducers

Significant factors for time to hepatotoxicity were liver metastasis (AHR 2.1, 95% CI 1.2–3.6) and CYP3A4 inducers (AHR 2.3, 95% CI 1.0–5.2) based on multivariate analysis (Table 5). As shown in Fig. 2, the mean time to hepatotoxicity in patients with and without CYP3A4 inducers was 598.9 and 1020.8 days (*p* = 0.075).

**Table 5:** Univariate and multivariate analyses to identify predictors for time to hepatotoxicity related to erlotinib administration

Characteristics	Unadjusted HR (95% CI)	Adjusted HR (95% CI)
Male	0.876 (0.530–1.448)	1.045 (0.579–1.886)
Age ≥ 65	1.314 (0.797–2.169)	1.265 (0.764–2.095)
BSA ≥ 1.6^a^	0.795 (0.483–1.309)	0.771 (0.431–1.377)
Liver metastasis	1.897 (1.100–3.275)^**^	2.052 (1.179–3.572)^*^
CYP3A4 inducer	2.017 (0.918–4.433)	2.318 (1.029–5.221)^*^

For multivariate analysis, factors with *p* < 0.1 in the univariate analysis were included in addition to sex, age and BSA

*BSA* body surface are

^a^There was 1 missing data for BSA

^*^P < 0.05, ^**^P < 0.01

Meanwhile, the median time to recovery from hepatotoxicity incidence was 71.0 days (range: 42.4~ 99.6 days). There was no significant factor on time to recovery in this study population.

## Discussion

We found that the concomitant use of CYP3A4 inducers increased hepatotoxicity 2.7-fold. In NSCLC patients, co-administration of H2-antagonist/PPI increased hepatotoxicity 3.5-fold. Among the demographic factors, liver metastasis and age ≥ 65 years were significant risk factors in all study patients and NSCLC patients, respectively; the attributable risk of liver metastasis and age was 46.3% and 71.8%, respectively. Subgroup analysis using pancreatic cancer patients yielded marginally significant results with CYP3A4 inducers and erlotinib-induced hepatotoxicity. Liver metastasis and CYP3A4 inducers also shortened time to hepatotoxicity 2.1 and 2.3-fold, respectively.

Erlotinib is extensively metabolized by CYP3A4 [7]. Concomitant use of CYP3A4 inducer reduces its exposure [9]; however, it increases the formation of metabolites, which may induce clinically adverse drug reactions. The mechanism of EGFR TKI-induced hepatotoxicity is not well-established. However, various EGFR TKIs have been shown to induce the formation of reactive metabolites leading to hepatotoxicity [10–12]. Erlotinib, one of the EGFR TKIs, is also oxidized to reactive epoxide and quinone-imine by cytochrome P450 [10]. In our previous study [13], CYP3A4 inducer was one of the significant factors underlying hepatotoxicity in NSCLC patients receiving gefitinib treatment in univariate analysis, although statistical significance was not observed in multivariate analysis. Similarly, combination of lapatinib and dexamethasone, one of the CYP3A4 inducers, showed 4.6-fold and 3.5-fold increased risk of hepatotoxicity and clinically important changes in ALT, respectively [14].

Pancreatic cancer patients showed higher hepatotoxicity incidence than NSCLC patients, consistent with results from another study [3, 6]. Concomitant use of gemcitabine chemotherapy was cited as one of the reasons for the higher incidence of hepatotoxicity in patients with pancreatic cancer. Gemcitabine also induces hepatotoxicity, although rarely [15].

Age 65 years and older was a risk factor for hepatotoxicity in the subgroup analysis of NSCLC patients, which contrasted with our previous gefitinib study correlating younger age with higher hepatotoxicity [13]. Considering that older individuals are usually more vulnerable to drug-induced diseases, the erlotinib result was not a surprise. In addition, other study supported this result; significantly higher rates of adverse drug reactions such as rash, tiredness, stomatitis, and dehydration were found in NSCLC patients aged 70 and above [16].

Erlotinib is known to be a substrate for adenosine triphosphate-binding cassette transporters (ABCB1, ABCG2, and ABCC10) [17]. ABCG2 and ABCB1 are expressed not only in tumor tissues but also in normal tissues including the liver [18]. PPIs and H2-antagonists are ABCG2 and ABCB1 inhibitors, which increase the concentration of ABCG2 and ABCB1 substrates such as erlotinib in the liver, resulting in hepatotoxicity. In our previous study on gefitinib, co-administration of PPIs and H2-antagonists showed significantly increased hepatotoxicity in NSCLC patients [13]. Another study evaluated the genetic polymorphism of ABC transporters on erlotinib-related adverse effects [19]. While the results did not reinforce the association between hepatotoxicity and genetic polymorphisms, they suggested that ABCG2 34G > A was a useful predictor of skin rash of grade 2 or higher level. Further, patients carrying ABCG2 -15,622 T/T polymorphism and ABCG2 (1143C/T, -15622C/T) haplotype developed significantly higher frequency of grade 2/3 diarrhea [20]. Based on the genetic results, ABCG2 represents a candidate marker of erlotinib-induced adverse reactions including hepatotoxicity.

The study limitations relate to the retrospective single-center design. In addition, EGFR mutation results were available only in NSCLC patients. Although patients with EGFR mutations manifested around 2.0-fold higher hepatotoxicity compared with those without mutation, no statistical significance was found (*p* = 0.379), possibly due to the small sample size (*n* = 155). However, to our knowledge, this is the first report to investigate the effect of concomitant drug use on erlotinib-induced hepatotoxicity. Also, this study is meaningful due to the large number of patients.

## Conclusions

In conclusion, our study showed that concomitant use of CYP3A4 inducers and H2-antagonist/PPI, liver metastasis, and age ≥ 65 were associated with erlotinib-induced hepatotoxicity. Thus, close monitoring of liver function is recommended, especially in patients using CYP3A4 inducers and anti-acid secreting agents.

## Abbreviations

AHR: Adjusted hazard ratio
ALT: Alanine aminotransferase
AOR: Adjusted odds ratio
AST: Aspartate aminotransferase
BSA: Body surface area
CTCAE: Common Terminology Criteria for Advanced Events
CYP: Cytochrome P450
ECOG: Eastern Cooperative Oncology Group
EGFR: Epidermal growth factor receptor
HR: Hazard ratio
NSCLC: Non-small cell lung cancer
OR: Odds ratio
PPI: Proton pump inhibitors
PS: Performance Status
TKI: Tyrosine kinase inhibitor
